# Editor's Letter-Box

**Published:** 1895-11-09

**Authors:** 


A. G. T. writes: The article entitled "The Fate of Colour-Blind Sailors," which appeared in your issue of the 2nd inst., deserves to be copied *in extenso* by the leading organs of the lay press. Mr. P. H. Bickerton has earned for himself the gratitude of the whole seafaring class (especially the lower grades) by his exposure of a state of things which should never have been possible to exist. It may interest some of your readers to note my brother's case. He, in company with an influential friend, sought the chairman of a leading line of mail steamers with a view to obtaining a berth as a junior officer on board one of their vessels. The sight test was applied, and my brother failed—this, after two years on a training ship, a four years' sea apprenticeship, and the obtaining of a second mate's certificate! The Board of Trade, we are told, apply no sight test till a candidate presents himself at a first mate's examination. The above occurred nearly three years ago. Needless to add, the whole of my brother's training was thrown away, and his life's prospects thus cruelly dashed to the ground.

